# Comparative Analysis of Rhizosphere and Endophytic Microbial Communities Between Root Rot and Healthy Root of *Psammosilene tunicoides*

**DOI:** 10.1007/s00284-023-03290-4

**Published:** 2023-05-18

**Authors:** Wen. T. Yang, Guo. D. Li, Jun. N. Li, Cheng. F. Yang, Xiao. M. Zhang, Ai. L. Zhang

**Affiliations:** Yunnan Provincial Key Laboratory of Molecular Biology for Sinomedicine, Yunnan University of Chinese Medicine, Yuhua Road, Chenggong District, Kunming, 650500 Yunnan People’s Republic of China

## Abstract

The wild resources of *Psammosilene tunicoides* have decreased sharply because of the long-term mining and excavation, which has led to the increased demand for its artificial cultivation. However, root rot represents a significant obstacle leading to a poor quality and product of *P. tunicoides*. Previous reports have not focused on root rot in *P. tunicoides.* Therefore, this study explores the rhizospheric and root endophytic microbial community structure and composition of healthy and root rot *P. tunicoides* to understand the mechanism underlying root rot*.* The properties of the rhizosphere soil were assessed using physiochemical methods, and the bacterial and fungal populations were studied through amplicon sequencing of the 16S rRNA genes and ITS regions in the root and soil. Compared to healthy samples, the pH, hydrolysis N, available P, and available K were significantly decreased in the diseased samples while the organic matter and total organic carbon were significantly increased in the diseased samples. Redundancy analysis (RDA) showed that soil environmental factors are related to changes in the root and rhizosphere soil microbial community of *P. tunicoides* indicating that the physiochemical properties of soil affect plant health. Alpha diversity analysis showed that the microbial communities of healthy and diseased samples were similar. Some bacterial and fungal genera were significantly increased or decreased (*P* < 0.05) in diseased *P. tunicoides*, and certain microbial factors that antagonized root rot were further explored. This study provides an abundant microbial resource for future studies and contributes to improving soil quality and *P. tunicoides* agricultural production.

**Supplementary Information:**

The online version contains supplementary material available at 10.1007/s00284-023-03290-4.

## Introduction

Recently, it has become increasingly acknowledged by researchers that endophytes found in medicinal plants, rhizosphere microorganisms, and other traditional Chinese medicine micro-ecological factors directly or indirectly affect the growth, properties, metabolism, and chemical composition of medicinal materials [1]. Among them, rhizosphere is the key microdomain of the interactions among the plant, soil and microorganism and also a place where probiotics and pathogens fight with each other [2]. The microecological rhizosphere is a soil microdomain that interacts with plant roots created by plants to improve plant health and growth [3]. The soil microbial flora is the most critical microecological factor affecting and regulating plant disease occurrences [4]. When plants are invaded by pathogenic bacteria, the rhizosphere microecology actively participates in regulating plant resistance systems by adjusting soil environmental factors and increasing the relative abundance of beneficial soil microorganisms or antagonistic antibacterial groups, thereby improving the soil environment, inhibiting pathogens, and improves plant disease resistance [5]. Raaijmakers et al. showed that a higher abundance of the antagonistic microbes strengthens the soil’s ability to inhibit soil-borne fungal diseases, and revealed that accumulation of soil-borne fungi pathogens enriches-specific rhizosphere microbial groups while causing soil-specific inhibition [2].

Additionally, there are many kinds of plant endophytes in nature. Previous studies have reported that endophytes have the same or similar anabolic pathways as host plants and thus can promote the growth and reproduction of plants. Endophytic bacteria can also improve the biological control ability of host plants by producing a large number of bioactive substances with novel chemical structures, good antibacterial effects or special effects. Changes in the internal and external environments of host plants will lead to changes in the rhizospheric soil and endophytic microbial communities [6]. Therefore, a better understanding the effects of the composition and function in the rhizosphere and endophytic microbial community will contribute to a better understanding of the mechanism underlying root rot diseased of plants and reveal new approaches for the rational use of plant-microbial interactions in agriculture.

*Psammosilene tunicoides* W.C.Wu et C.Y.Wu (Caryophyllaceae) is a narrowly distributed and endemic plant species in southwestern China and represents an ancient and famous Asian herbal medicine. The active ingredient of *P. tunicoides* is an oleanane-type triterpene saponin that has analgesic, anti-inflammatory, immune regulatory, bactericidal, and antibacterial properties [7]. The occurrence of root rot has seriously affected the quality and yield of *P. tunicoides* medicinal materials and restricted the development and utilization of *P. tunicoides* resources by enterprises, such as Yunnan Baiyao. We conducted field trips to several major cultivation areas of *P. tunicoides* in Yunnan (Yuxi, Dali, Qujing, and others), China, and found that two or three-year-old *P. tunicoides* are prone to root rot during the rainy season from July to August. Typical symptoms of *P. tunicoides* root rot include shrinking of the root epidermis, intensification of dark yellow color, and softening of the roots after the color turns black-brown. Thereafter, the root begins to rot slowly moves upwards from the tip of the root. Based on these preliminary visits, we found that root rot does not occur in one-year-old *P. tunicoides* and mainly occurs starting in the second to third year. However, diseased root samples are difficult to collect in the third year because the diseased roots will slowly rot away over time. Thus, farmers generally harvest one-year-old *P. tunicoides* for fear of losses caused by the diseases, which results in a medicinal extract that does not reach pharmaceutical standards due to the insufficient cultivation time. However, systematic reports are not available on root rot pathogen species of *P. tunicoides*. Therefore, this study analyzed *P. tunicoides* root rot disease and healthy rhizosphere microecological characteristics, including the soil physiochemical properties, root and rhizosphere soil microflora diversity, and structure and potential functions of the rhizosphere microecology. The key microecological factors affecting root rot were probed, and the regulatory mechanism of the rhizosphere microecology on root rot in *P. tunicoides* was explored to provide a scientific basis for its prevention and control.

## Materials & Methods

### Study Site and Sample Collection

This study was conducted at the Jianchuan *P. tunicoides* cultivation base in Dali, Yunnan Province, on November 1, 2020. The annual average temperature is 11–22 ℃, and the average annual precipitation is 757.6–880.0 mm. The soil of the *P. tunicoides* cultivation base is loose and fertile loam, which provides good growing conditions for *P. tunicoides*. At this site, the roots and rhizosphere soil of two-year-old healthy and diseased *P. tunicoides* were collected.

First, the topsoil was removed (10–15 cm), and then healthy and diseased *P. tunicoides* root samples were gently gathered from a depth of 20 cm using a sterile shovel, with care taken to keep the root system intact. Large clumps of soil on the roots were removed, rhizosphere soil was collected by brushing off soil attached to 1–2 mm roots. A total of 24 specimens were obtained, with 12 specimens each from the root and soil samples. Six duplicates were collected for each of the healthy and diseased sample groups, respectively. Each soil sample was passed through a 2 mm sieve and placed in a sterile tube, and then rinsed three times with distilled water, for surface disinfection. Samples of each tissue type tissue were immersed in 70% ethanol for 4 min and then washed three times with sterile distilled water. The root samples also were surface disinfected in the same way, after washing with running tap water to remove the attached soil. Then, all samples were homogenized in sterile plastic bags and shipped to the laboratory, where they were frozen at − 80 °C until further analysis (a total of twenty four samples). Amplicon sequencing of bacterial 16S rRNA genes and fungal ITS regions of twelve *P. tunicoides* root samples and twelve rhizosphere soil samples were tested, among them, twelve rhizosphere soil samples were also used for the physical and chemical properties test.

### Determination of Soil Physiochemical Properties

The physical and chemical properties of six healthy soil samples and six diseased soil samples were tested, and the content of conventional elements in the soil was detected. The electrode method was used to detect the soil pH value (water-soil ratio 1:1; NY/T1377-2007), and the dichromic acid oxidation-external heating method was used to detect the soil organic matter (NY/T 1121.6-2006). The distillation method was used to detect hydrolysis N (LY/T1228-2015), the Olsen method was used to detect available P (NY/T 1121.7–2014), and the ammonium acetate extraction-flame photometric method was used to detect available K (NY/T889-2004).

### DNA Extractions

DNA was extracted using the CTAB method according to the instructions. The reagent designed that was used to extract DNA from trace samples has been shown to be effective in preparing DNA from most bacteria. Nuclear-free water was used as a blank in the experiments. The total DNA was eluted in 50 μL elution buffer and stored at − 80 °C until polymerase chain reaction (PCR) assays were performed at LC-Bio Technology Co., Ltd. (Hangzhou, Zhejiang Province, China).

### PCR Amplification and 16S rRNA and ITS Sequencing

The 5' ends of the primers were tagged with two primers: specific DNA barcodes (for each sample) and universal sequencing primers. PCR amplification was performed using a 25 μL reaction mixture, which contained 25 ng of template DNA, 12.5 μL DNA polymerase (2X Phanta Max master mix), 2.5 μL of each primer, and PCR-grade water to adjust the volume. The PCR conditions to amplify the prokaryotic 16S and ITS fragments consisted of initial denaturation at 98 ℃ for 30 s; 32 cycles of denaturation at 98 ℃ for 10 s, annealing at 54 ℃ for 30 s, and extension at 72 ℃ for 45 s; and then a final extension at 72 ℃ for 10 min. The specific primer set 341F (5'-CCTACGGGNGGCWGCAG-3')/805R(5'-GACTACHVGGGTATCTAATCC-3'), ITS1FI2 (5'-GTGARTCATCGAATCTTTG-3')/ITS2 (5'-TCCTCCGCTTATTGATATGC-3') with barcode sequences was used in the PCR-amplify the V3-V4 regions of the bacterial 16S rRNA genes (468 bp product), and the ITS2 regions of fungi, respectively. The PCR products were identified using 2% agarose gel electrophoresis. In the PCR amplification procedure, ultrapure water was employed as a negative control template to verify the absence of false-positive amplification events. The PCR products were purified using AMPure XT beads (Beckman Coulter Genomics, Danvers, MA, USA) and quantified using Qubit (Invitrogen, USA). The amplicon pools were used for sequencing, and the size and quantity of the amplicon library were evaluated using the Agilent 2100 Bioanalyzer (Agilent, USA) and the Illumina Library Quantitative Kit (Kapa Biosciences, Woburn, MA, USA). The libraries were sequenced on NovaSeq PE250 platform.

### Data Analysis

The samples were sequenced on an Illumina NovaSeq platform according to the manufacturer's recommendations, as provided by LC-Bio. Paired-end reads were assigned to the samples based on their unique barcodes, and they were truncated using cutadapt (v1.9) [8] to cut off the barcode and primer sequence. The paired-end reads were merged using FLASH (v1.2.8) and PEAR (v0.9.6) [9, 10]. According to the fqtrim (v0.94) [11]. Quality filtering on the raw reads was performed under specific filtering conditions to obtain high-quality clean tags. Thereafter, Vsearch software (v2.3.4) [12] was used to filter chimeric sequences, and DADA2 [13] and the concept of amplicon sequence variants (ASVs) were used to construct a table for operational taxonomic units (OTUs) [14]. Finally, a feature table and feature sequence were obtained. Alpha diversity and beta diversity were normalized by flattening, in which the same number of sequences were extracted randomly by reducing the number of sequences to the minimum of some samples. Species were annotated using the relative abundance for normalization (X flora count/total count). Both the alpha and beta diversity were calculated using QIIME2 [15], and graphs were constructed using R package (v3.5.2) [16]. The complexity of species diversity for a sample was assessed using three alpha diversity indices: Chao1, Shannon, Simpson. Beta diversity analyses usually starts by calculating the distance matrix between environmental samples, which includes the distance between any two samples. Differences between samples were determined based on principal component analysis (PCA) and clustering analysis (UPGMA). Kruskal–Wallis or Mann Whitney *U* tests, were implemented to test for significant differences (*P* < 0.05) between the variances of microbial communities. RDA was performed to consider the influence of soil environmental factors, and it can simultaneously reflect the relationship between samples, environmental factors, and species. Other diagrams were implemented using the R package (v3.5.2) [16]. BLAST was used for sequence alignment, and the feature sequences were annotated with the SILVA database (release 132) [17, 18] and UNITE databases [19].

## Results

### Rhizosphere Soil Physiochemical Properties

The soil physicochemical properties of six healthy and six diseased rhizosphere soils collected from the *P. tunicoides* cultivation area were determined. The characteristics of the soil samples showed that the pH, hydrolysis N, available P, and available K of the diseased samples decreased considerably compared to that of the healthy samples. In contrast, the organic matter and total organic carbon contents of the diseased samples were significantly higher than those of the healthy samples (Table 1 [*P* < 0.05]).

**Table 1:** Physical and chemical nature of rhizospheric soil

			Test items			
Sample name	pH	Organic matter	Hydrolysis N	Available P	Available K	Total organic carbon
	Water-soil ratio = 2.5:1	g/kg	mg/kg	mg/kg	mg/kg	g/kg
Healthy	5.60 ± 0.05	24.27 ± 1.53	135.91 ± 27.12	62.44 ± 10.04	417.87 ± 76.13	14.05 ± 0.89
Diseased	5.17 ± 0.09^**^	55.35 ± 3.36^***^	56.10 ± 4.59^**^	29.69 ± 6.33^*^	208.33 ± 25.60^**^	31.99 ± 1.91^*^

Data are expressed as means ± standard deviation (six copies each of healthy and diseased rhizosphere soil samples)

*Significant effect at *p* ≤ 0.05 level

**Significant effect at *p* ≤ 0.01 level

***Significant effect at *p* ≤ 0.001 level

### Microbial Community Diversity in the Root and Rhizosphere Soil of *P*. *tunicoides*

**Fig. 1:**
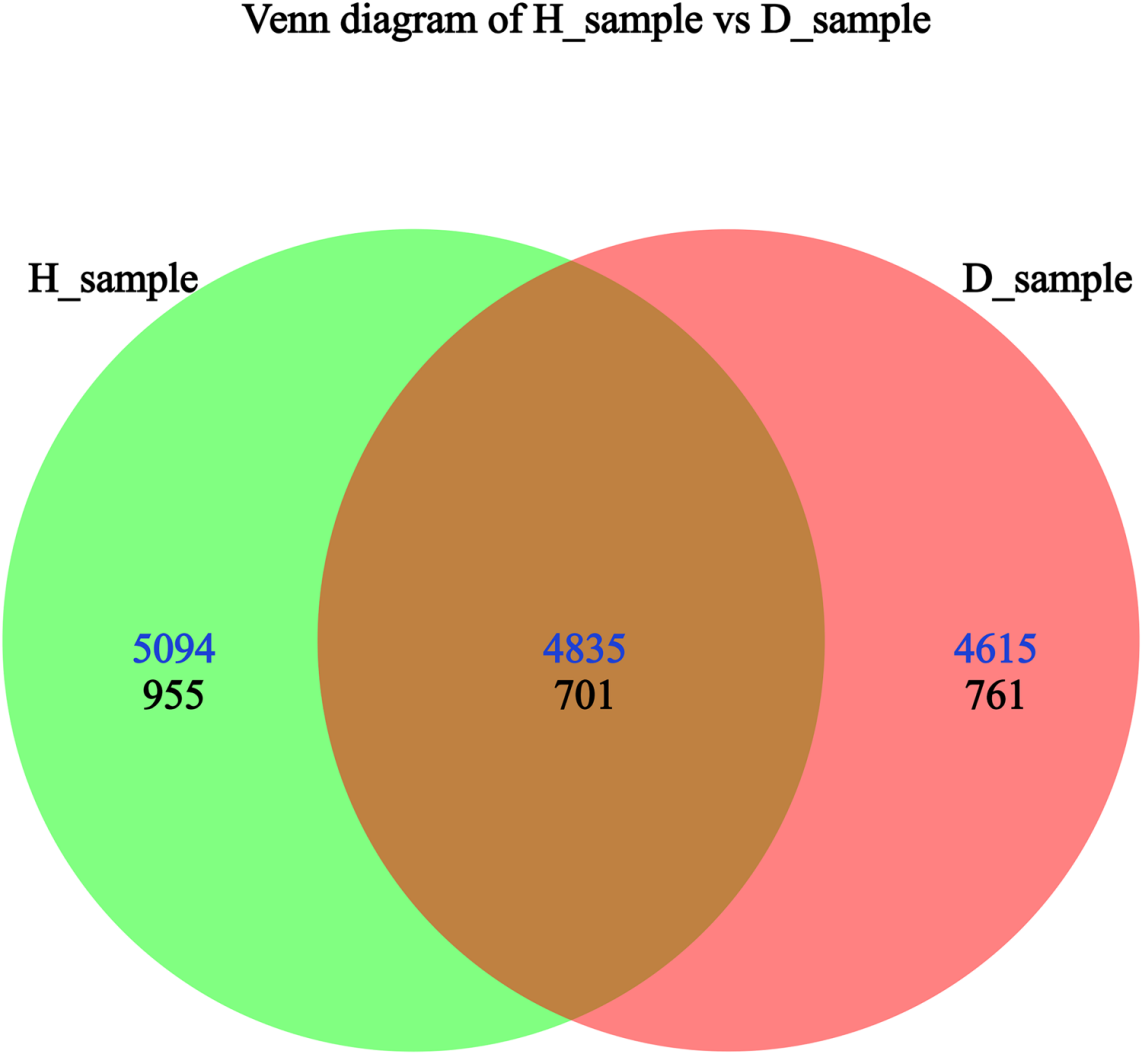
Venn diagrams of shared bacterial (blue numbers) and fungal (black numbers) OTUs in root and rhizosphere soil of *P. tunicoides*

Libraries containing the V3-V4 hypervariable region of bacterial 16S rRNA gene fragments and fungal ITS2 sequences were separately constructed. The entire bacterial sequencing data set from the root and rhizosphere soil samples of healthy plants and diseased plants contained 2,022,854 raw reads, and 1,870,658 high-quality sequences, which indicated that 92.48% of the sequences passed the stringent quality control and filtering processes. The fungal sequencing data contained 2,030,195 raw reads and 1,990,950 high-quality sequences (98.07%, Table 2). A total of 4,615 bacterial OTUs and 761 fungal OTUs were identified in diseased plants; 5,094 bacterial OTUs and 955 fungal OTUs were identified in healthy plants; and the percentage of shared OTUs between healthy and diseased samples was lower for the fungal community than the bacterial communities (Fig. 1, Table S2). This indicates that large numbers of bacteria and fungi are present in the roots and rhizosphere soil of healthy and diseased *P. tunicoides*. All of these sequences were subjected to taxonomy-based analyses. Bacterial and fungal sequences were assigned to 40,840 and 5,947 OTUs, respectively. Among them, the rhizosphere soil contained the highest number of OTUs (Table 2).

**Table 2:** Quality control of high-throughput sequences and the OTU assignment

Sample		Raw reads	Valid reads	OTUs	Genera
Fungi	Root	1,014,468	999,992	798	113
	Rhizosphere soil	1,015,727	990,958	5149	368
Bacteria	Root	1,013,993	941,822	9781	652
	Rhizosphere soil	1,008,861	928,836	31,059	794

Richness (Chao1 index) and diversity indices (Shannon index and Simpson index) of the microbial community and the number of OTUs in the root and rhizosphere soil samples from healthy and diseased P. tunicoides are shown in Table S1. Among them, the Goods coverage index approached 1 for all samples, indicating that the sequencing depth covered all species. In general, the alpha diversity of the bacteria and fungi in the root and rhizosphere soil were similar between the healthy and diseased samples, indicating that their community composition was similar (Figure S2). The healthy and diseased samples in the roots and rhizosphere soil did not completely cluster using the UPGMA when the taxa and samples were sorted according to the abundance distribution of taxa or the degree of similarity between samples using the clustering results (Figure S1).

PCA was performed according to the different OTU compositions of all samples in the root and rhizosphere soil using the zero baseline of the two coordinate axes as a reference (Figure S1). PCA1 and PCA2 of the bacterial communities in the root explained 53.9% and 17.83% of the differences, respectively, while PCA1 and PCA2 of the bacterial communities in the rhizosphere soil explained 73.35% and 15.86% of the differences, respectively. Meanwhile, PCA1 and PCA2 of the fungal communities in the roots explained 51.59% and 27.67% of the differences, respectively, and PCA1 and PCA2 of the fungal communities in the rhizosphere soil explained 61.37% and 23.88% of the differences, respectively. These results are consistent with the clustering graph, and the healthy and diseased group root and rhizosphere soil samples were not obviously clustered together.

### Root and Rhizosphere Soil Microbial Community Compositions of Healthy and Diseased *P*. *tunicoides*

**Fig. 2:**
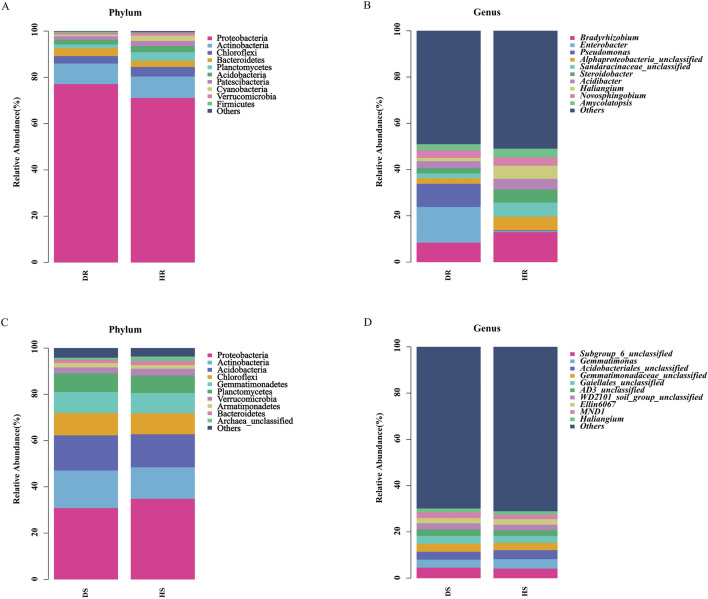
Relative abundance of bacterial phyla and genera in healthy (H) and diseased (D) groups. A and C: Bacterial phylum of root and rhizosphere soil, respectively; B and D: bacterial genus of root and rhizosphere soil, respectively

In the bacterial communities of the roots, Proteobacteria was the most abundant bacterial phyla and accounted for 77.09% and 71.14% of the root bacteria from diseased and healthy plants, respectively. Meanwhile, Rokubacteria and Tenericutes were only present in diseased plants while FCPU426, Hydrogenedentes, Kiritimatiellaeota, and FBP were only present in healthy plants (Fig. 2A). At the genus level, the most abundant genus in diseased plants was *Enterobacter* (15.50%), which only represented 0.40% of the bacterial population in healthy plants. The most abundant genus in healthy plants was *Bradyrhizobium* (12.95%) (Fig. 2B). In rhizosphere soil, Proteobacteria represented the most abundant bacteria from diseased plants (30.83%) and healthy plants (34.86%) (Fig. 2C). The taxonomic abundance of dominant bacteria at the genus level was not obvious, and the 10 most abundant genera of bacteria are presented in Fig. 2D.

**Fig. 3:**
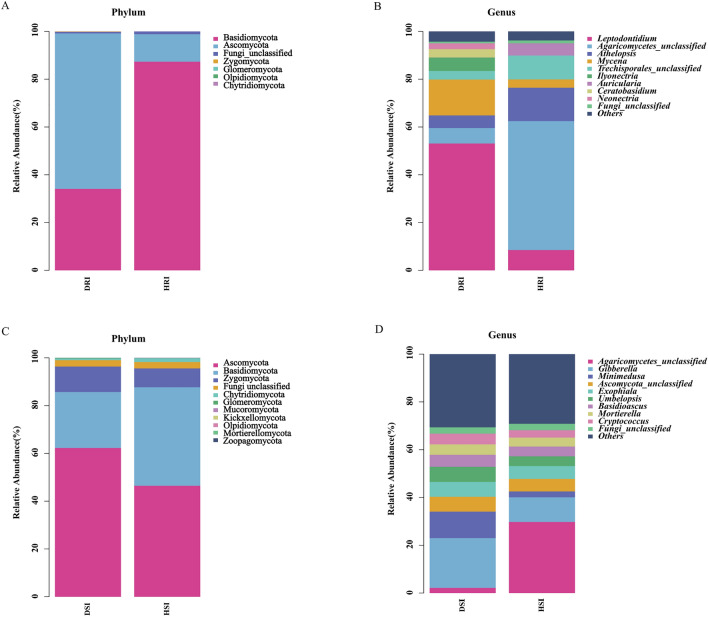
Relative abundance of fungal phyla and genera in healthy (H) and diseased (D) groups. A and C: Fungal phyla of root and rhizosphere soil, respectively; B and D: fungal genera of root and rhizosphere soil, respectively

The most abundant fungal phyla in diseased plant roots were Ascomycota (65.10%), followed by Basidiomycota, unclassified Fungi, Zygomycota, Glomeromycota, and Olpidiomycota. Meanwhile, the most abundant phyla in healthy plants were Basidiomycota (87.37%), followed by Ascomycota, unclassified Fungi, Zygomycota, and Chytridiomycota (Fig. 3A). The most abundant genera detected in diseased and healthy plants were *Leptodontidium* (53.08%) and unclassified Agaricomycetes (53.95%) (Fig. 3B). Unclassified Lyophyllaceae and *Nectriaceae* only exist in diseased plants, while *Auricularia* and unclassified Chaetothyriales only exist in healthy plants. Ascomycota was the most abundant fungal phyla in rhizosphere soil of diseased samples (62.30%) and healthy samples (46.46%), respectively(Fig. 3C). The most abundant genera from diseased and healthy samples were *Gibberella* (20.79%) and unclassified Agaricomycetes (29.73%), respectively after analyzing the 10 most abundant genera (Fig. 3D).

### Analysis of Significant Differences

**Fig. 4:**
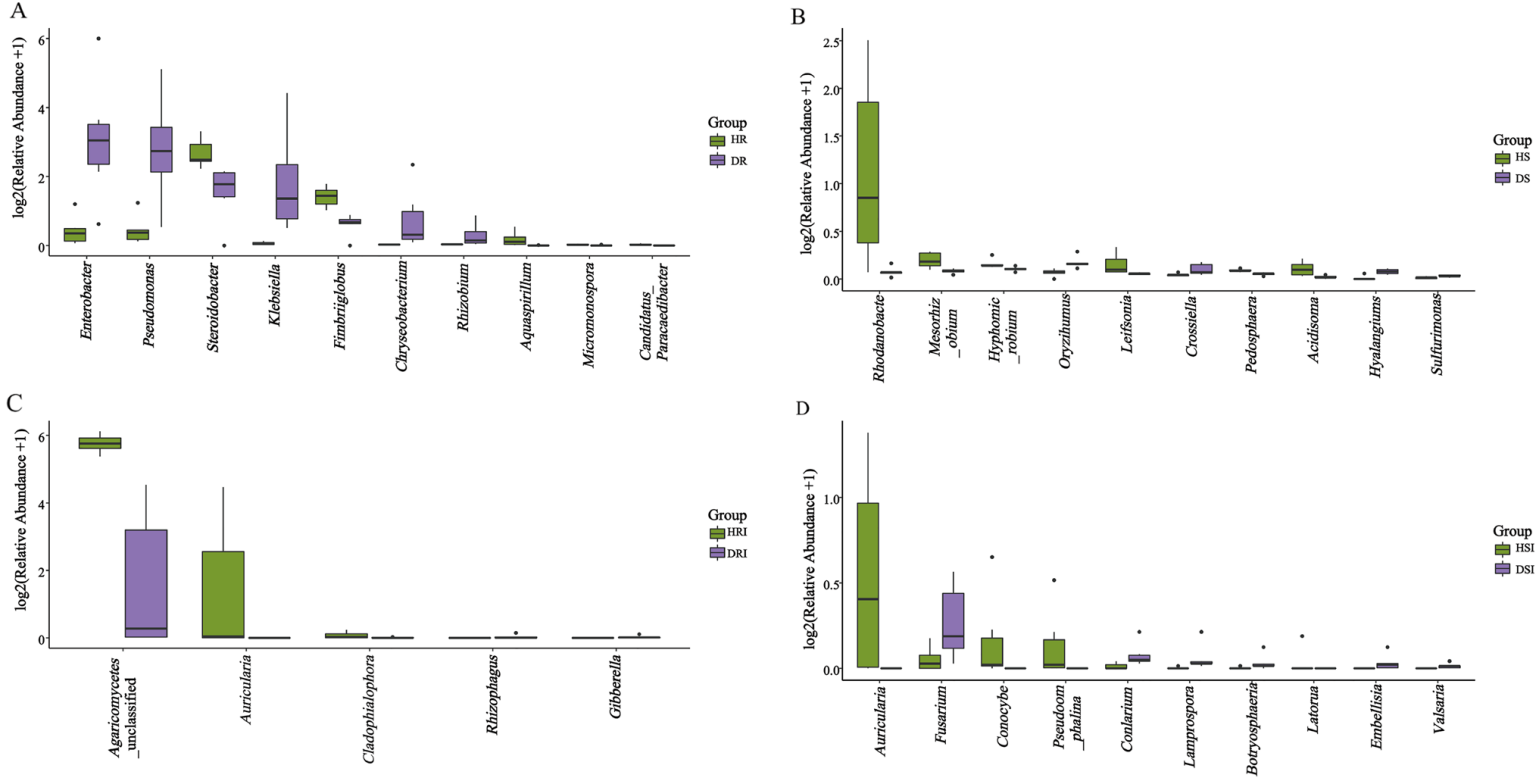
Significant differences between biomarker species from healthy (H) and diseased (D) groups. A and B: Bacterial species from the root and rhizosphere soil, respectively; C and D: fungal species from the root and rhizosphere soil, respectively. When performing log2 processing on data, a value of 1 was added to the valid data first, and then log2 processing was performed to avoid calculating log2(0)

Significant differences were found across the different biomarker species between the healthy and diseased *P. tunicoides* roots and rhizosphere soils using the species difference test (ANOVA, *P* < 0.05; Fig. 4). In the roots, there are 42 bacterial genera with significant differences, such as *Enterobacter*, *Pseudomonas*, unclassified Alphaproteobacteria, unclassified Sandaracinaceae, *Steroidobacter* and *Haliangium,* which were included in the top 10. There were six fungal genera with significant differences, among which the unclassified Agaricomycetes and *Auricularia* were included in the top 10. In the rhizosphere soil, there were 68 bacterial genera with significant differences, although these genera were not in the top 10, and there were 19 fungal genera with significant differences, with only *Minimedusa* in the top 10 (ANOVA, *P* < 0.05; Table S3).

### RDA Analysis

**Fig. 5:**
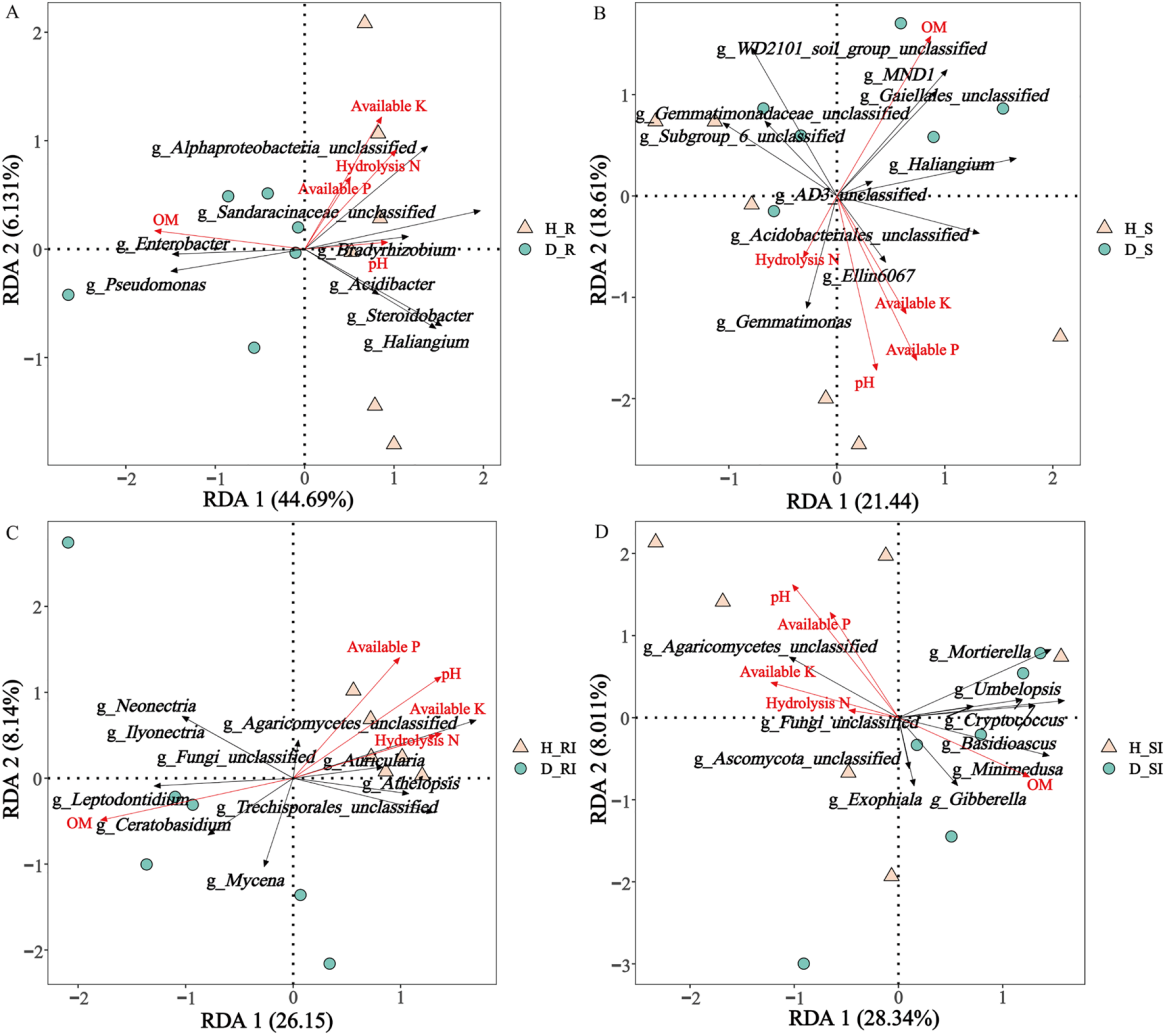
RDA analysis of *P. tunicoides* rhizosphere microbial community and soil factors. A and B: RDA analysis of bacterial communities and soil factors in root and rhizosphere soils, respectively; C and D: RDA analysis of fungal communities and soil factors in root and rhizosphere soils, respectively. Triangles, circles, and red arrows represent healthy samples, diseased samples, and soil properties, respectively

The top 10 genera of microorganisms from healthy and diseased *P. tunicoides* root and rhizosphere soil samples were selected for RDA analysis with the soil physicochemical properties to determine their relationship with the rhizosphere microbial community. The majority of soil environmental factors (pH, hydrolysis N, available P, and available K) were found on the same side as healthy root and rhizosphere soil samples, indicating that root health is positively affected by these soil properties (Fig. 5). In contrast, the organic matter and total organic carbon contents were found on the same side as the diseased samples.

In the root, over 50.82% of the bacterial community variation in the *P. tunicoides* root was explained by 2 canonical axes, with RDA 1 and RDA 2 accounting for 44.69% and 6.13% of the variation, respectively. Unclassified Alphaproteobacteria, unclassified Sandaracinaceae, *Bradyrhizobium*, *Acidibacter*, *Steroidobacter*, and *Haliangium* were positively correlated with environmental factors (pH, hydrolysis N, available P, and available K), indicating that *P. tunicoides* health status was positively affected by these bacteria. *Enterobacter* and *Pseudomonas* were negatively correlated with these environmental factors and found on the same side as the diseased samples, indicating that they had a negative impact on *P. tunicoides* health. Over 34.29% of the fungal community variation was explained by 2 canonical axes, with RDA 1 and RDA 2 accounting for 26.15% and 8.14% of the variation, respectively. Unclassified Agaricomycetes, *Athelopsis*, unclassified Trechisporales, *Auricularia*, and unclassified Fungi were positively correlated with environmental factors (pH, hydrolysis N, available P, and available K). Meanwhile, *Leptodontidium*, *Mycena*, *Ilyonectri*a, *Ceratobasidium*, and *Neonectria* were negatively correlated with these environmental factors.

In the rhizosphere soil, over 40.05% of the bacterial community variation was explained by 2 canonical axes, with RDA 1 and RDA 2 accounting for 21.44% and 18.61% of the variation, respectively. Unclassified Acidobacteriales, *Gemmatimonas*, and *Ellin6067* were positively correlated with most environmental factors (pH, hydrolysis N, available P, and available K). Meanwhile, unclassified Subgroup_6, unclassified Gemmatimonadaceae, unclassified Gaiellales, unclassified AD3, unclassified WD2101_soil_group, *MND1*, and *Haliangium* were negatively correlated with these environmental factors. Over 36.35% of the fungal community variation was explained by 2 canonical axes, with RDA 1 and RDA 2 accounting for 28.34% and 8.01% of the variation, respectively. Unclassified Agaricomycetes was positively correlated with most environmental factors (pH, hydrolysis N, available P, and available K), while *Gibberella*, *Minimedusa*, unclassified Ascomycota, *Exophiala*, *Umbelopsis*, *Basidioascus*, *Mortierella*, *Cryptococcus*, and unclassified Fungi were negatively correlated with these environmental factors.

## Discussion

In this study, a comprehensive and detailed comparison of the physicochemical properties of healthy rhizosphere soil and diseased rhizosphere soil was conducted to investigate the relationship between *P. tunicoides* root rot and soil properties. The pH of diseased *P. tunicoides* rhizosphere soil significantly decreased compared with that of the healthy rhizosphere soils. The soil pH has specific effects on plant growth and soil microbial community structure, diversity, and richness [3, 20]. Therefore, disease-associated fungi or bacteria may appear in soils as the pH changes. Ji et al. showed that the pH in soil samples obtained from diseased American ginseng plants was lower than that in soil samples obtained from healthy American ginseng plants [21], which is consistent with our results. In addition, phosphorus (P) is an important component of the plant cell nucleus and membrane. Normal P levels contribute to the healthy metabolism of plant protein, stimulate plant root growth, increase mineral nutrient absorption by rhizomes, and reduce the damage caused by plant diseases [22]. Potassium (K) promotes the development of the thick outer wall of epidermal cells and prevents the occurrence of disease [22, 23]. Rousk et al. found that the decline in pH values after continuous cropping of *Aconitum carmichaeli* could probably inhibit nitrogen and potassium absorption by plants, resulting in the occurrence of soil-borne pathogens [24]. In our results, the available P and available K content of diseased rhizosphere soils with an decreased pH was significantly lower than that of healthy rhizosphere soils. Overall, root rot may be related to reduce plant disease resistance caused by lower pH, available P, available K, and hydrolysis N in the soil. Our survey showed that cultivated *P. tunicoides* was susceptible to diseases during cultivation; therefore, pesticides were sprayed to control the diseases both in healthy and diseased plants. Spraying pesticides will lead to the acidification of soil for both healthy and diseased *P. tunicoides.* Thus, we considered that the rhizospheric soil in *P. tunicoides* cultivation areas would be slightly acidic compared to the soil of wild *P. tunicoides* areas, and such conditions are not suitable for the growth of *P. tunicoides*. Meanwhile, we found that the organic matter and total organic carbon contents were significantly higher in diseased samples. Our field survey showed that *P. tunicoides* mainly grows in the wild in barren environments, such as rock crevices; however, *P. tunicoides* is usually cultivated using farmyard manure as a base fertilizer before sowing, with organic fertilizer applied post-sowing. These *P. tunicoides* plants have excellent yield and quality, although the excess organic matter and total organic carbon may cause root rot. Li et al. also found that the organic matter and total organic carbon in the diseased rhizosphere soil were higher than those in healthy soil, which may be related to the organic fertilizer applied by farmers during planting [25], which is consistent with our findings. In further studies, we will provide *P. tunicoides* planting suggestions for farmers based on our results.

Bian et al. found that certain soil environmental factors contribute to changes in the rhizosphere microbial community by RDA analysis [26]. In our study, the RDA analysis also indicated that the health of *P. tunicoides* was positively affected by pH, available P, available K, and hydrolysis N, and negatively affected by organic matter and total organic carbon. Furthermore, our RDA results revealed correlations between several beneficial or pathogenic microorganisms’ microbial taxa in the roots and rhizosphere soil of *P. tunicoides* and environmental variables.

The Chao1, Shannon, and Simpson indices and OTU number indicated that the healthy samples and diseased samples were similar. In addition, the PCA and clustering results showed that the healthy and diseased groups were not obviously clustered together. In many previous studies, the microbial community diversity of healthy plants was always higher than that of diseased plants, such as in the *Panax ginseng* [24], *Aconitum carmichaeli* [26], and *Panax notoginseng* [27]. However, our results showed that the Chao1, Shannon, and Simpson indices of healthy samples and diseased samples were similar. Thus, the same agronomic management and cultivation environment, such as spraying pesticides, affects the microbial community diversity of healthy plant of *P. tunicoides*, which results in a similar level of diversity in both healthy and diseased plants. Fang et al. found that pesticides might directly inhibited the reproduction of certain rhizosphere microorganisms during ginseng cultivation, which results in a decrease in microbial diversity of farmland-cultivated ginseng [28]. Rosenblueth et al. proposed that changes in the number and types of microorganisms in the rhizosphere and root surface can lead to *Rhizobium etli* maize [29]. Gao et al. showed that the plant microbial community determines plant growth and development. Overall, changes in microbial community structures impact *P. tunicoides* health.

The stacked bar chart showed that Proteobacteria was the predominant microbial phylum in healthy and diseased samples and showed widespread occurrence in the growing environment of *P. tunicoides*; however, the relative abundance of this phylum varied widely between the roots and rhizosphere soil. The proportion of Proteobacteria in the healthy and diseased groups was 71.14% and 77.09% in the roots, respectively, and 34.86% and 30.83% in the rhizosphere soil, respectively. Proteobacteria contains many pathogenic bacteria, including *Escherichia*, *Helicobacter*, and others [30]. Actinobacteria was also a predominant group in healthy and diseased samples, and the proportion of Actinobacteria in the rhizosphere soil was greater than that in the root. Actinobacteria are gram-positive, mostly saprophytic bacteria that are generally distributed in the soil, and some are parasitic bacteria that can cause disease [31]. Yang et al. found that Acidobacteria abundance is significantly negatively correlated with the infection rate of *Nicotiana tabacum* L. bacterial wilt [32]. In addition, in our study, we found that Rokubacteria and Tenericutes were only present in diseased plants, and these bacterial genera may play an important role in *P. tunicoides* root rot. Moreover, FCPU426, Hydrogenedentes, Kiritimatiellaeota, and FBP were only present in healthy plants.

At the genus level, there are differences in some bacterial community genera composition between the healthy and diseased group of *P. tunicoides* roots, diseased plants contain a higher abundance of *Enterobacter* (15.50%), *Pseudomonas* (9.94%), and *Klebsiella* (4.95%), while these genera only represented 0.40%, 0.42%, and 0.05% in healthy plants, and the abundance of all genera was significantly diminished. RDA analysis showed that *Enterobacter* and *Pseudomonas* were negatively correlated with most soil environmental factors (pH, hydrolysis N, available P, and available K), which shows that they play an important role in *P. tunicoides* root rot. *Klebsiella* is pathogenic bacteria of plants [33], some species of *Enterobacter* and *Pseudomonas* are also pathogenic bacteria, such as *Enterobacter cloacae* can cause *ginger* root rot, *Pseudomonas cerasi* is a pathogen of wild cherry [34, 35]. These results provide insights for exploring the causes of *P. tunicoides* disease. Interestingly, Chernin et al. found that *Enterobacter* suppresses the growth of different pathogenic fungi [36], while *Pseudomonas* is involved in bacteriostasis of root rot, has plant colonization characteristics, and exhibits antagonistic properties against soil-borne plant pathogenic fungi [37]. The most abundant healthy plant bacterial genera in *P. tunicoides* was *Bradyrhizobium* (12.95%). The RDA analysis performed in this work showed that *Bradyrhizobium* was negatively correlated with most soil environmental factors (pH, hydrolysis N, available P, and available K), which is consistent with previous studies showing that it belongs to the genus of beneficial bacteria [38].

However, the taxonomic abundance of the dominant bacterial genera in rhizosphere soil was not obvious. For example, the abundance of *Rhodanobacter* was significantly increased in healthy rhizosphere soil and had an antagonistic effect on the *Fusarium* fungal pathogen that causes *ginseng* root rot [39], indicating that this bacterium may have an inhibitory effect on *P. tunicoides* disease and is potentially involved in biocontrol. In short, the changes in the roots and rhizosphere soil bacterial community structure of *P. tunicoides* changed the plant’s health and caused root rot. The determination of antagonistic bacteria provides abundant microbial flora resources to control *P. tunicoides* root rot.

Fungi play an important role in the *P. tunicoides* ecosystem. At the phylum level, there is a certain similarity between healthy samples and diseased samples. Ascomycota, Basidiomycota, Zygomycota, and Glomeromycota exist in both the healthy group and the diseased group, and their compositions are similar between the two groups; however, their abundance differs. In roots, Basidiomycota (87.37%) is the most abundant phylum in healthy plants while Ascomycota is the most abundant phylum in diseased plants (65.10%). Glomeromycota was detected in roots and soil. In roots, Glomeromycota was only present in the diseased samples; while, in rhizosphere soil, the content of disease samples was higher than that of the healthy samples. Its aggregate bacteria can form arbuscular mycorrhizae (AM) of terrestrial plants; thus, it is called “AM fungi” [40]. AM has an important ecological significance for the growth and survival of most vascular plants [41]. Bennett et al. found that AM fungi can exert controlling effects on plant defensive phenotypes [42]. However, Eck et al. revealed that AM fungi introduce both benefits and risks to host plant. Host plants inoculated with AM fungi not only showed increased growth in every population, but also increased the infection rate of a fungal pathogen (*Podosphaera plantaginis*) to host [43]. Mendes et al. mentioned that when attacked by pathogens or insects, plants are able to recruit protective microorganisms and enhance microbial activity to suppress pathogens in the rhizosphere [44]. Therefore, diseased plants could recruit AM fungi to help plants resist diseases and grow better. In this study, Ascomycota had the highest abundance of dominant phyla in root rot samples (62.30%) and healthy samples (46.46%) in rhizosphere soil. Ascomycota exists in terrestrial, marine, and freshwater habitats, and many species play an important ecological role as decomposers. Ascomycota is a mostly terrestrial species that may utilize saprophytic, parasitic, and symbiotic nutritional methods. Saprophytic Ascomycetes can cause mildew of wood, food, cloth, and leather and decompose animal and plant residues [45]. Therefore, Ascomycota may be involved in *P. tunicoides* pathogenesis.

In addition, differences in the fungal genera were observed between the healthy and diseased groups, with unique fungal genera in each group. Unclassified Lyophyllaceae and *Nectriaceae* were only observed in diseased *P. tunicoides* root samples, thus showing that some of the unique fungal groups may exhibit pathogenicity and cause disease. Meanwhile, *Auricularia* and unclassified Chaetothyriales were only observed in healthy samples. The most abundant genera detected in the roots of root rot samples was *Leptodontidium* (53.08%). *Leptosphaeria* is a high- temperature and high-humidity pathogen that can infect rape and cause black shank disease [46]. Our results showed that *Gibberella* was the dominant genus in diseased rhizosphere soil samples and was negatively correlated with most soil environmental factors (pH, hydrolysis N, available P, and available K) by RDA analysis. Some species of *Gibberella* are important soil borne pathogen, and many *Gibberella* species can cause devastating plant diseases, such as *Fusarium* head blight [47]. Moreover, some species of this genus were also a great producer of Gibberellins (GA, plant hormone). GA has a variety of regulatory functions on plant growth and can promote stem elongation, induce plant flowering, break seed dormancy, and enhance plant resistance [48]. Furthermore, the fungal pathogen *Fusarium* showed significant differences in rhizosphere soil. Guo R et al. found that the main pathogen causing *Panax notoginseng* root rot is *Fusarium* [49]. The dominant genus in the root and rhizosphere soil of healthy samples was unclassified Agaricomycetes, and differences in abundance were observed.

Thus, the occurrence of *P. tunicoides* root rot is related to the composition of the fungal community in the roots and rhizosphere soil. The composition and diversity of fungal communities in healthy and diseased samples were similar, but there were increases in fungal population diversity were observed in the diseased group. Pang et al. found that the beneficial flora in the plant and rhizosphere soil decreased during plant growth, and pathogen proliferation may cause atrophy, blackening, rotting, and other symptoms of the root epidermis [50]. The increase in the relative abundance of pathogenic fungi within *P. tunicoides* likely led to *P. tunicoides* root rot. Therefore, the change in fungal community structure is one of the important reasons for *P. tunicoides* root rot.

## Conclusions

In our previous investigation, we found that root rot diseased is very serious in the cultivated areas of *P. tunicoides* in Yunnan, thus affecting the production of certain enterprises in Yunnan. We carried out the investigation of root rot of *P. tunicoides* in various cultivated areas of Yunnan. First, *P. tunicoides* mainly grows in barren environments such as rock crevices and saline-alkali soil under our wild survey. However, we found that *P. tunicoides* is usually cultivated in slightly acidic soil or soil with excess organic matter to achieve fast growth and high yield, which is one of the reasons for the root rot of *P. tunicoides.* Therefore, in our subsequent work, we will provide plant suggestions to farmers for the cultivation of *P. tunicoides* based on our results*.* Second, high-throughput sequencing technology was used to determine the diversity, structure, potential functions of microbial colonies in the root and rhizosphere soil of *P. tunicoides*. During cultivation, pesticides were sprayed to control the diseases, which is one of the reasons for the decrease of microbial community diversity of soil. Thus, in this study, the functions of some microbial factors in antagonizing root rot were explored which contributes to revealing the cause of this disease. *Rhodanobacter* and *Pseudomonas* are examples of biocontrol bacteria in the *P. tunicoides* rhizosphere. Therefore, it is necessary to screen microorganisms with inhibitory effects on pathogens in the rhizosphere to provide a theoretical basis for the effective use of plant microecological control measures to prevent *P. tunicoides* root rot. Future studies should focus on identifying fungal and bacterial genera against root rot pathogens, which will control disease and contribute to improving *P. tunicoides* agricultural production.

## Supplementary Information

Below is the link to the electronic supplementary material.

Supplementary file1 (TIF 19277 KB) UPGMA clustering analyses and PCA of high-throughput sequencing amplicons of healthy and diseased P. tunicoides. A and B: Bacterial UPGMA clustering analyses and PCA of root; C and D: bacterial UPGMA clustering analyses and PCA of rhizosphere soil; E and F: fungal UPGMA clustering analyses and PCA of root; G and H: fungal UPGMA clustering analyses and PCA of rhizosphere soil.

Supplementary file2 (TIF 20347 KB) Alpha diversity indices. A, B and C: Bacterial Chao1, Shannon, and Simpson indices of root, respectively; D, E and F: bacterial Chao1, Shannon, and Simpson indexes of rhizosphere soil, respectively; G, H and I: fungal Chao1, Shannon, and Simpson indices of root, respectively; and J, K and L: fungal Chao1, Shannon, and Simpson indices of rhizosphere soil, respectively.

Supplementary file3 (DOCX 19 KB)

## Data Availability

All raw sequences acquired in this study have been submitted to the Sequence Read Archive (SRA) of the NCBI under accession number PRJNA873035 (https://www.ncbi.nlm.nih.gov/sra/ PRJNA873035).

## References

[1] Nalini MS, Prakash HS, Tejesvi MV, Egamberdieva D, Tiezzi A (2019). Bioactive potentials of novel molecules from the endophytes of medicinal plants. Medically important plant biomes source of secondary metabolites.

[2] Raaijmakers JM, Paulitz TC, Steinberg C, Alabouvette C, Moënne-Loccoz Y (2009). The rhizosphere: a playground and battlefield for soilborne pathogens and beneficial microorganisms. Plant Soil.

[3] Wang S, Cheng J, Liao Y (2020). Response of rhizosphere microecology to plants, soil and microbes. Acta Microsc.

[4] Thuerig B, Fließbach A, Berger N, Fuchs JG, Kraus N, Mahlberg N, Nietlispach B, Tamm L (2009). Re-establishment of suppressiveness to soil- and air-borne diseases by re-inoculation of soil microbial communities. Soil Biol Biochem.

[5] Somers E, Vanderleyden J, Srinivasan M (2004). Rhizosphere bacterial signalling: a love parade beneath our feet. Crit Rev Microbiol.

[6] Tan Y, Cui Y, Li H, Kuang A, Li X, Wei Y, Ji X (2017). Diversity and composition of rhizospheric soil and root endogenous bacteria in *Panax notoginseng* during continuous cropping practices. J Basic Microbiol.

[7] Zhang XY, Tang YJ, Zhou MC, Xu QZ, Li HQ (2013). Advances in medicinal plant *Psammosilene*
*tunicoides*. Guizhou Agric Sci.

[8] Martin M (2011). cutadapt removes adapter sequences from high-throughput sequencing reads. EMBnet J.

[9] Mago T, Salzberg SL (2011). FLASH: fast length adjustment of short reads to improve ge-nome assemblies. Bioinformatics.

[10] Zhang JJ, Kobert K, Flouri T, Stamatakis A (2014). PEAR: a fast and accurate illumina paired-end reAd mergeR. Bioinformatics.

[11] Pertea G (2018) gpertea/fqtrim: Fqtrim Release (Version v0.9.7). 10.5281/zenodo.1185412

[12] Rognes T, Flouri T, Nichols B, Quince C, Mahe F (2016). VSEARCH: a versatile open source tool for metagenomics. PeerJ.

[13] Callahan BJ, McMurdie PJ, Rosen MJ, Han AW, Johnson AJA, Holmes SP (2016). DADA2: High-resolution sample inference from Illumina amplicon data. Nat Methods.

[14] Blaxter M, Mann J, Chapman T, Thomas F, Whitton C, Floyd R, Abebe E (2005). Defining operational taxonomic units using DNA barcode data, philosophical transactions of the royal society of london. Philos Trans R Soc B.

[15] Bolyen E, Rideout JR, Dillon MR (2019). Reproducible, interactive, scalable and extensible microbiome data science using QIIME 2. Nat Biotechnol.

[16] Dixon P (2003). VEGAN, a package of R functions for community ecology. J Veg Sci.

[17] Glöckner FO, Yilmaz P, Quast C, Gerken J, Beccati A, Ciuprina A, Bruns G, Yarza P, Peplies J, Westram R, Ludwig W (2017). 25 years of serving the community with ribosomalRNA gene reference databases and tools. J Biotechnol.

[18] Quast C, Pruesse E, Yilmaz P, Gerken J, Schweer T, Yarza P, Peplies J, Glöckner FO (2012). The SILVA ribosomal RNA gene database project: improved data processing andweb-based tools. Nucleic Acids Res.

[19] Henrik NR, Karl-Henrik L, Taylor Andy FS, Johan BP, Jeppesen TS, Dmitry S, Peter K, Kathryn P, Oliver GF, Leho T (2019). The UNITE database for molecular identification of fungi: handling dark taxa and parallel taxonomic classifications. Nuclc Acids Res.

[20] Guo JH, Liu XJ, Zhang Y, Shen JL, Han WX, Zhang WF, Christie P, Goulding KWT, Vitousek PM, Zhang FS (2010). Significant acidification in major chinese croplands. Science.

[21] Ji L, Nasir F, Tian L, Chang J, Sun Y, Zhang J, Li X, Tian C (2021). Outbreaks of root rot disease in different aged american ginseng plants are associated with field microbial dynamics. Front Microbiol.

[22] Dordas C (2009). Role of nutrients in controlling plant diseases in sustainable agriculture. A review. Agron Sustain Agric.

[23] Sharma S, Duveiller E, Basnet R, Karki CB, Sharma RC (2005). Effect of potash fertilization on Helminthosporium leaf blight severity in wheat, and associated increases in grain yield and kernel weight. Field Crop Res.

[24] Rousk J, Bååth E, Brookes PC, Lauber CL, Lozupone C, Caporaso JG, Knight R, Fierer N (2010). Soil bacterial and fungal communities across a pH gradient in an arable soil. ISME J.

[25] Li L, Xiang D, Wu YF, Huang YD, Li H, Zhang XM, Liang B (2022). Effects of long-term different fertilization patterns on soil nutrients and microbial community structure of tomato in a solar greenhouse. Ying Yong Sheng tai xue bao = J Appl Ecol.

[26] Xia F, Wang LN, Chen JY, Fu M, Wang GD, Yan YP, Cui LJ (2021). Variations of microbial community in *Aconitum*
*carmichaeli* Debx. rhizosphere soilin a short-term continuous cropping system. J Microbiol.

[27] Tan Y, Cui Y, Li H, Kuang A, Li X, Wei Y, Ji X (2017). Rhizospheric soil and root endogenous fungal diversity and composition in response to continuous *Panax notoginseng* cropping practices. Microbiol Res.

[28] Fang X, Wang H, Zhao L, Wang M, Sun M (2022). Diversity and structure of the rhizosphere microbial communities of wild and cultivated *ginseng*. BMC Microbiol.

[29] Rosenblueth M, Martínez-Romero E (2004). Rhizobium etli maize populations and their competitiveness for root colonization. Arch Microbiol.

[30] Ahrens A, Lipski A, Klatte S, Busse HJ, Auling G, Altendorf K (1997). Polyphasic classification of *Proteobacteria* isolated from biofilters. Syst Appl Microbiol.

[31] Ventura M, Canchaya C, Tauch A, Chandra G, Fitzgerald GF, Chater KF, van Sinderen D (2007). Genomics of *Actinobacteria*: tracing the evolutionary history of an ancient phylum. Microbiol Mol Biol Rev.

[32] Yang H, Li J, Xiao Y, Gu Y, Liu H, Liang Y, Liu X, Hu J, Meng D, Yin H (2017). An integrated insight into the relationship between soil microbial community and tobacco bacterial wilt disease. Front Microbiol.

[33] Podschun R, Ullmann U (1998). *Klebsiella* spp. as nosocomial pathogens: epidemiology, taxonomy, typing methods, and pathogenicity factors. Clin Microbiol Rev.

[34] Zhao N, Yang J, Liu H, Li L, Yan H, Liu D (2022). Ginger Rhizome Rot caused by the *Enterobacter* cloacae in Tangshan. China Plant Dis.

[35] Ilii R, Jelui A, Markovi S, Baracet G, Bagi F, Popovic T (2021). *Pseudomonas cerasi*, the new wild cherry pathogen in Serbia and the potential use of recG helicase in bacterial identification. Ann Appl Biol.

[36] Chernin L, Ismailov Z, Haran S, Chet I (1995). Chitinolytic *Enterobacter* agglomerans antagonistic to fungal plant pathogens. Appl Environ Microbiol.

[37] Lan DY, Shu FL, Lu YH, Shou AF, Lin W, Yuan GQ (2022). First report of a leaf spot disease of tobacco caused by *Pseudomonas cichorii* in China. Plant Dis.

[38] Antoun H, Beauchamp CJ, Goussard N, Chabot R, Lalande R (1998). Potential of rhizobium and bradyrhizobium species as plant growth promoting rhizobacteria on non-legumes: effect on radishes (*raphanus*
*sativus* l.). Plant Soil.

[39] Huo Y, Kang JP, Park JK, Li J, Chen L, Yang DC (2018). *Rhodanobacter ginsengiterrae* sp. nov., an antagonistic bacterium against root rot fungal pathogen *Fusarium solani*, isolated from *ginseng* rhizospheric soil. Arch Microbiol.

[40] Wang XF, Zhang X, Zhao RH, Yu J, Gu W, Li R, Cao GH, He S (2020). Effect and mechanism of arbuscular mycorrhizal fungi in herbs. Chin J Exp Tradit Med Formulae.

[41] Abdellatif L, Lokuruge P, Hamel C (2019). Axenic growth of the arbuscular mycorrhizal fungus Rhizophagus irregularis and growth stimulation by coculture with plant growth-promoting rhizobacteria. Mycorrhiza.

[42] Bennett AE, Bever JD, Deane BM (2009). Arbuscular mycorrhizal fungal species suppress inducible plant responses and alter defensive strategies following herbivory. Oecologia.

[43] Eck JL, Kytöviita MM, Laine AL (2022). Arbuscular mycorrhizal fungi influence host infection during epidemics in a wild plant pathosystem. New Phytol.

[44] Mendes R, Garbeva P, Raaijmakers JM (2013). The rhizosphere microbiome: significance of plant beneficial, plant pathogenic, and human pathogenic microorganisms. FEMS Microbiol Rev.

[45] Liu W, Sun C (2021). C 17 -fengycin B, produced by deep-sea-derived *Bacillus subtilis*, possessing a strong antifungal activity against *Fusarium solani*. J Oceanol Limnol.

[46] Brachaczek A, Kaczmarek J, Jedryczka M (2021). Warm and wet autumns favour yield losses of oilseed rape caused by phoma stem canker. Agronomy.

[47] Vigier B, Reid LM, Dwyer LM (2001). Maize resistance to gibberella ear rot: symptoms, deoxynivalenol, and yield1. Can J Plant Pathol.

[48] Kawaide H (2006). Biochemical and molecular analyses of gibberellin biosynthesis in fungi. Biosci Biotechnol Biochem.

[49] Guo R, Liu X, Li S, Miao Z (2009). In vitro inhibition of fungal root-rot pathogens of *Panax notoginseng* by rhizobacteria. Plant Pathol J.

[50] Pang Z, Mao X, Xia Y, Xiao J, Wang X, Xu P, Liu G (2022). Multiomics reveals the effect of root rot on *Polygonati Rhizome* and identifies pathogens and biocontrol strain. Microbiol Spectrum.

