# Supplementary figures and images for: Comparative Analysis of Rhizosphere and Endophytic Microbial Communities Between Root Rot and Healthy Root of Psammosilene tunicoides
Source: Curr Microbiol. 2023 May 18;80(7):215. doi: 10.1007/s00284-023-03290-4 (PMC10191990; doi:10.1007/s00284-023-03290-4)

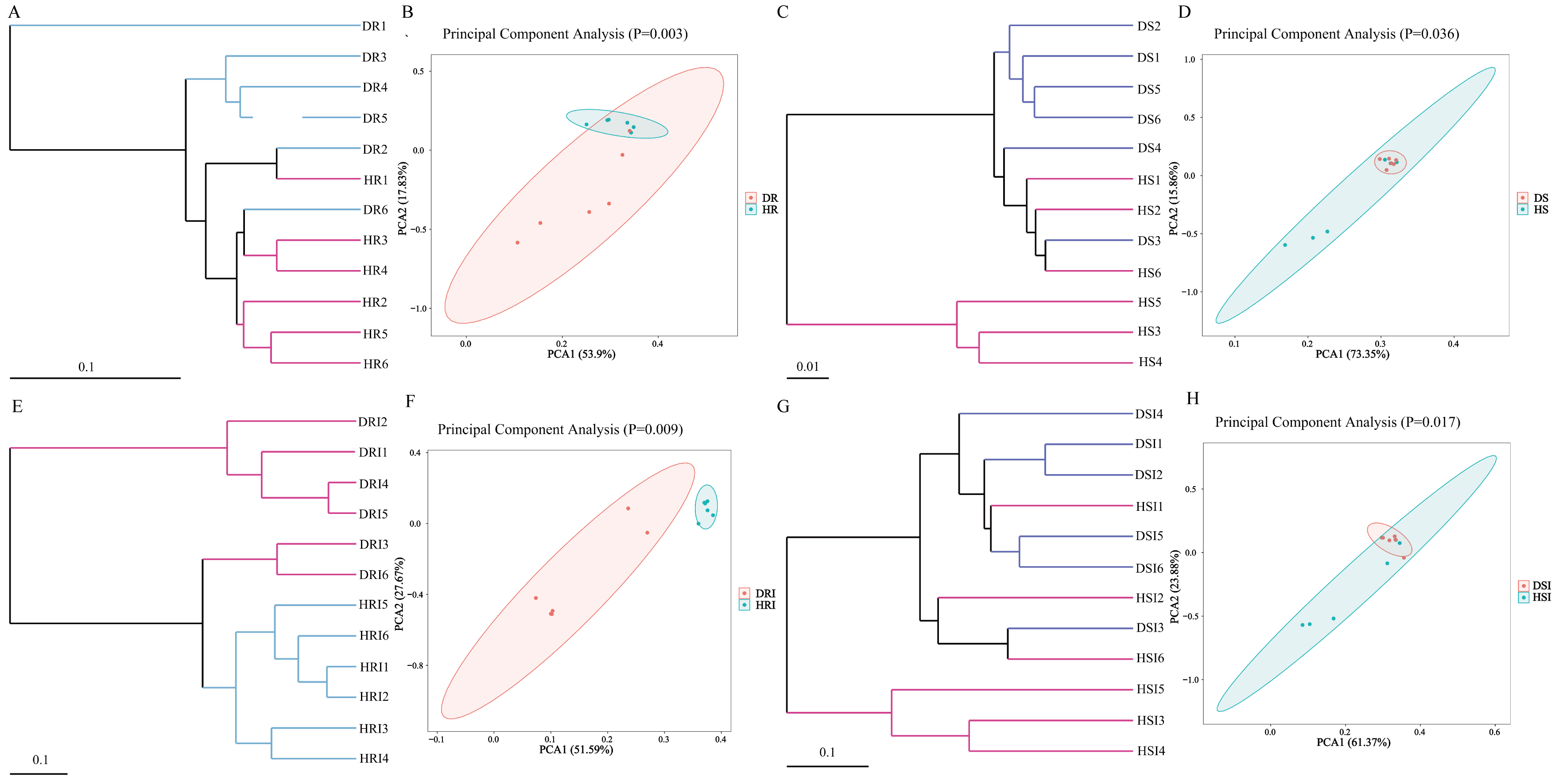

Supplement: Supplementary file 1 — Supplementary file1 (TIF 19277 KB) UPGMA clustering analyses and PCA of high-throughput sequencing amplicons of healthy and diseased P. tunicoides. A and B: Bacterial UPGMA clustering analyses and PCA of root; C and D: bacterial UPGMA clustering analyses and PCA of rhizosphere soil; E and F: fungal UPGMA clustering analyses and PCA of root; G and H: fungal UPGMA clustering analyses and PCA of rhizosphere soil. [file 284_2023_3290_MOESM1_ESM.tif]

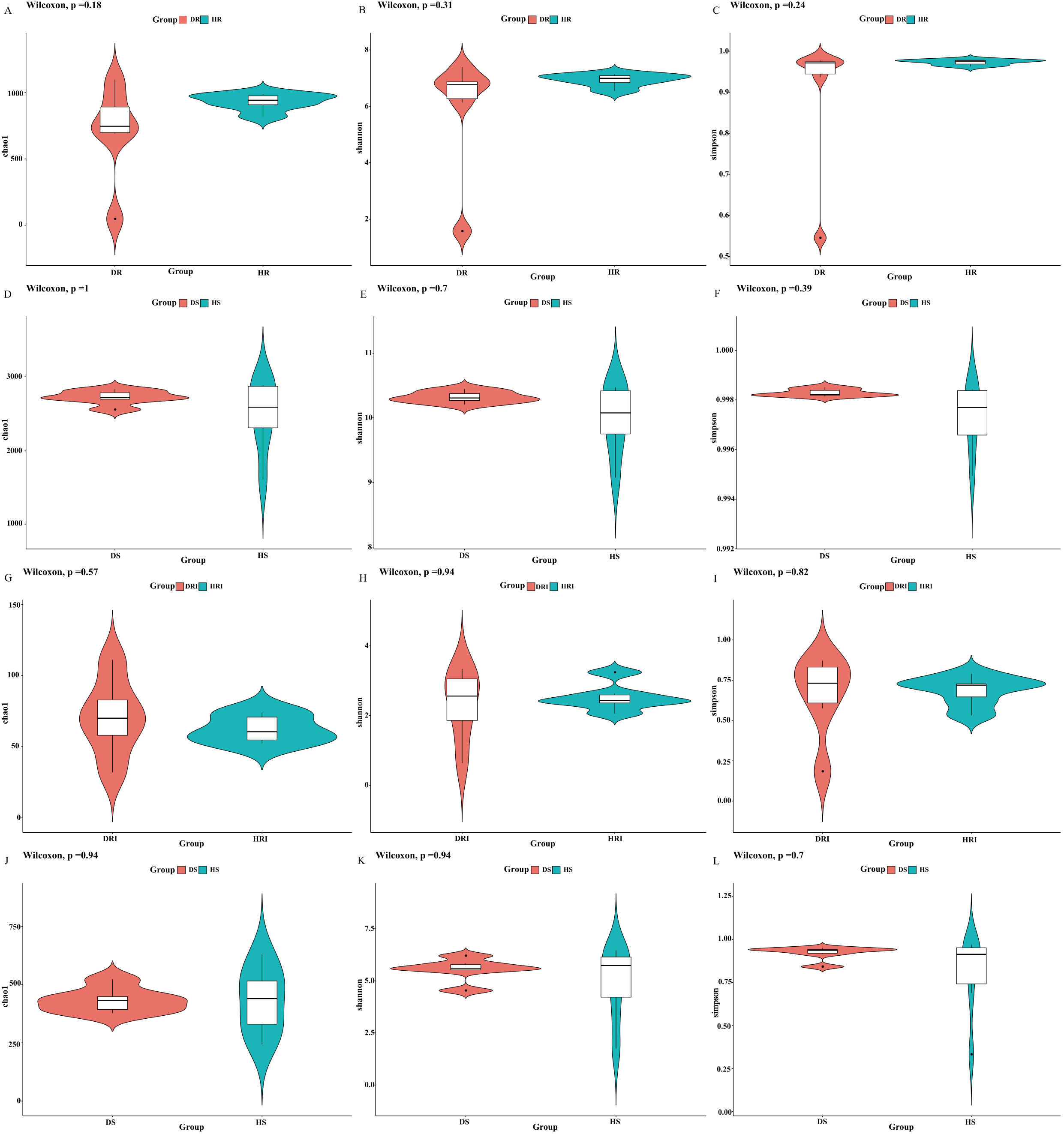

Supplement: Supplementary file 2 — Supplementary file2 (TIF 20347 KB) Alpha diversity indices. A, B and C: Bacterial Chao1, Shannon, and Simpson indices of root, respectively; D, E and F: bacterial Chao1, Shannon, and Simpson indexes of rhizosphere soil, respectively; G, H and I: fungal Chao1, Shannon, and Simpson indices of root, respectively; and J, K and L: fungal Chao1, Shannon, and Simpson indices of rhizosphere soil, respectively. [file 284_2023_3290_MOESM2_ESM.tif]
