# Supplementary material for: Comparative Analysis of Rhizosphere and Endophytic Microbial Communities Between Root Rot and Healthy Root of Psammosilene tunicoides
Source: Curr Microbiol. 2023 May 18;80(7):215. doi: 10.1007/s00284-023-03290-4 (PMC10191990; doi:10.1007/s00284-023-03290-4)
Supplement: Supplementary file 3 — Supplementary file3 (DOCX 19 KB) [file 284_2023_3290_MOESM3_ESM.docx]

**Supplementary tables**

**Comparative analysis of rhizosphere and endophytic microbial communities between root rot and healthy root of *Psammosilene tunicoides***

Wen. T Yang^1†^, Guo. D. Li^1†^, Jun. N. Li^1^, Cheng. F. Yang^1^, Xiao. M. Zhang^1*^, Ai. L.Zhang^1*^

^1^ Yunnan Provincial Key Laboratory of Molecular Biology for Sinomedicine (Yunnan University of Chinese Medicine), Kunming 650500, P. R. China.

Authors:

Wenting Yang

Email address: yangwentingmn@126.com

ORCID: https://orcid.org/0000-0003-0383-6842

Guodong Li

Email address: [gammar116@163.com](mailto:gammar116@163.com)

ORCID: https://orcid.org/0000-0002-9108-5454

Junnan Li

Email address: [1640797904@qq.com](mailto:1640797904@qq.com)

ORCID: https://orcid.org/0000-0002-8433-4408

Chengfan Yang

Email address: 643409227@qq.com

ORCID: [https://orcid.org/0000-0002-8841-7](https://orcid.org/0000-0002-1213-6998)147

Corresponding Authors:

Aili Zhang

Yunnan University of Chinese Medicine, Yuhua Road, Chenggong District, Kunming 650500, Yunnan, P. R. China

Email address: [yunnanzhongyi@hotmail.com](mailto:yunnanzhongyi@hotmail.com)

ORCID: https://orcid.org/0000-0002-6169-6537

Xiaomei Zhang

Yunnan University of Chinese Medicine, Yuhua Road, Chenggong District, Kunming 650500, Yunnan, P. R. China

Email address: 14022@ynutcm.edu.cn

ORCID: https://orcid.org/0000-0002-2681-5844

^†^These authors contributed equally to this work.

**^*^**These corresponding authors contributed equally to this work.

**Table S1** Alpha diversity indices in the root and rhizosphere soil of health and disease *P. tunicoides* samples

| Sample | | Health | | |  |  | Disease | | |  |
| --- | --- | --- | --- | --- | --- | --- | --- | --- | --- | --- |
|  |  | Richness index | Diversity index | |  |  | Richness index | Diversity index | |  |
|  |  | chao 1 | shannon | simpson | OTUs |  | chao 1 | shannon | simpson | OTUs |
| fungi | Root | 62.33 | 2.52 | 0.69 | 374 |  | 70.67 | 2.32 | 0.66 | 424 |
|  | Rhizosphere soil | 431.08 | 4.94 | 0.80 | 2555 |  | 433.19 | 5.55 | 0.92 | 2594 |
| bacteria | Root | 929.05 | 6.93 | 0.97 | 5543 |  | 710.16 | 5.92 | 0.90 | 4238 |
|  | Rhizosphere soil | 2467.86 | 9.98 | 1.00 | 14791 |  | 2713.09 | 10.32 | 1.00 | 16268 |

**Table S2** Numbers at the six classification levels of all samples

| Samples | | Root | Rhizosphere soil |
| --- | --- | --- | --- |
| Bacteria | Phylum | 31 | 42 |
|  | Class | 96 | 127 |
|  | Order | 197 | 269 |
|  | Family | 340 | 439 |
|  | Genus | 652 | 794 |
|  | Species | 866 | 1093 |
| Fungi | Phylum | 7 | 11 |
|  | Class | 19 | 42 |
|  | Order | 46 | 100 |
|  | Family | 81 | 205 |
|  | Genus | 112 | 368 |
|  | Species | 139 | 530 |

**Table S3** Different species (genus) with significant differences in different *P. tunicoides* samples

| Sample | | Up | Down |
| --- | --- | --- | --- |
| Bacteria | Root  (27 of 42 in total) | *Candidatus_Paracaedibacter*  *Aquaspirillum*  *Steroidobacter*  *Fimbriiglobus*  *Micromonospora*  *Rhodocista*  *Dactylosporangium*  *Gluconobacter*  *Plot4-2H12*  *Holophaga*  *Singulisphaera*  *Haliangium*  *Burkholderia*  *Telmatospirillum*  *Thermosporothrix*  *Solwaraspora* | *Klebsiella*  *hryseobacterium*  *Enterobacter*  *Pseudomonas*  *Rhizobium*  *Massilia*  *Ensifer*  *Gaiella*  *Paenarthrobacter*  *Serratia*  *Stenotrophomonas* |
|  | Rhizosphere soil  (39 of 68 in total) | *Leifsonia*  *Mesorhizobium*  *Pedosphaera*  *Rhodanobacter*  *Hyphomicrobium*  *Acidisoma*  *Sphaerobacter*  *Nitrolancea*  *Rhodopseudomonas*  *Phenylobacterium*  *Asticcacaulis*  *Holophaga*  *Jeongeupia*  *Cryocola*  *Mizugakiibacter*  *Actinocatenispora*  *Pseudolabrys*  *Dokdonella*  *Actinoplanes*  *FCPS473*  *Caulobacter*  *Chryseolinea*  *GAS113* | *Oryzihumus*  *Sulfurimonas*  *Hyalangium*  *Crossiella*  *Saccharopolyspora*  *MND1*  *Sulfurifustis*  *Anaeromyxobacter*  *Iamia*  *Geodermatophilus*  *Candidatus_Koribacter*  *Fontimonas*  *Dactylosporangium*  *Aciditerrimonas*  *Jatrophihabitans*  *Kibdelosporangium* |
| Fungi | Root  (4 of 5 in total) | *Auricularia*  *Cladophialophora* | *Rhizophagus*  *Gibberella* |
|  | Rhizosphere soil  (17 of 19 in total) | *Conocybe*  *Pseudoomphalina*  *Auricularia*  *Latorua*  *Cotylidia*  *Glomerella* | *Lamprospora*  *Conlarium*  *Embellisia*  *Valsaria*  *Botryosphaeria*  *Fusarium*  *Ochroconis*  *Minimedusa*  *Eupenicillium*  *Rhinocladiella*  *Paraglomus* |
